# Supplementary material for: Quantification and optimization of platinum–molybdenum carbide interfacial sites to enhance low-temperature water-gas shift reaction
Source: Nat Commun. 2025 Jan 28;16:1098. doi: 10.1038/s41467-025-55886-y (PMC11775272; doi:10.1038/s41467-025-55886-y)
Supplement: Supplementary file 1 — Supplementary Information [file 41467_2025_55886_MOESM1_ESM.pdf]

Supplementary Information

Quantification and optimization of platinum–molybdenum carbide interfacial sites to enhance low-temperature water-gas shift reaction

Ruiying Li [1], Jingyuan Shang [1], Fei Wang [1], Qing Lu [1], Hao Yan [1], Yongxiao Tuo [1], Yibin Liu [1]*, Xiang Feng [1]*, Xiaobo Chen [1], De Chen [2]*, Chaohe Yang [1]*

[1]State Key Laboratory of Heavy Oil Processing, China University of Petroleum, Qingdao, Shandong 266580, P.R. China;

[2]Department of Chemical Engineering, Norwegian University of Science and Technology, Trondheim 7491, Norway;

Corresponding authors

Correspondence to: Yibin Liu (liuyibin@upc.edu.cn); Xiang Feng (xiangfeng@upc.edu.cn); De Chen (de.chen@ntnu.no); Chaohe Yang (yangch@upc.edu.cn)

Supplementary note 1

Could the Pt be embedded in the carbide substrate?

Firstly, the thickness of the Pt atomic layer can be determined by comparing the thickness of the Pt atomic layer with the line profile intensity of the isolated atoms (Fig. 1(d4), Fig. 1(e4), and Fig.S7-S8). The Pt atoms are dispersed in a single atomic layer on the α-MoC_{1-x} surface.

Secondly, the coordination numbers of Pt with C and Mo on Pt_1/α-MoC-111-C_{vac}, Pt_1/α-MoC-111-Mo_{vac}, Pt_1/α-MoC-111, Pt_1/α-MoC-100-C_{vac}, Pt_1/α-MoC-100-Mo_{vac}, Pt_1/α-MoC-100 models were counted (as shown in Fig. S20). The results showed that the coordination number of Pt embedded in carbide substrate increased significantly. On the 0.2% Pt/α-MoC_{1-x} catalyst with single-atom dispersion, the coordination numbers of Pt-C and Pt-Mo are lower than those of the embedded model (Pt_1/α-MoC-111-C_{vac}, Pt_1/α-MoC-111-Mo_{vac}, and Pt_1/α-MoC-100-Mo_{vac}).

In summary, we infer that Pt atom is not embedded in the carbide substrate.

Supplementary note 2

Why was the CO molecule chosen as the probe molecule for quantitative analysis of cluster size and active sites?

The spillover phenomenon refers to the phenomenon that the active center (original active center) on the surface of the solid catalyst produces an ionic or free radical active species through adsorption, and they migrate to other active centers (secondary active centers). They can induce new activity or carry out certain chemical reactions through chemical adsorption. Firstly, the DFT calculations and previous literature research conclusions show that CO molecules are adsorbed on the surfaces of both Pt metal and α-MoC_{1-x} support (Fig. S3), which suppress CO spillover effects on the Pt/catalyst surface.

Secondly, the adsorption behavior of CO and H at different sites was analyzed by DFT calculation. As shown in Fig. S3 and Table S1, the CO adsorption configurations on the α-MoC-111, α-MoC-100, Pt/α-MoC-111, Pt/α-MoC-100, and Pt-111 are all linear, and adsorption energies at different sites are slightly different. Both Pt and Mo sites have strong adsorption strength for CO molecules. As shown in Fig. S2 and Table S1, there are two configurations of H adsorption on different surfaces: three-coordinate adsorption and linear adsorption. It is worth noting that the adsorption energy of H at the Pt site on the Pt_1/α-MoC-111 model and the Pt_1/α-MoC-100 model is positive, which means that the adsorption strength is weak. This variation in H adsorption configuration makes it challenging to accurately quantify Pt and Mo sites. Therefore, calculating the cluster size of Pt by the difference in the adsorption amount of CO on different catalysts is the most feasible solution.

Third, the H_2-O_2 titration experiment is also not feasible. The reason is that α-MoC_{1-x} is highly sensitive in the presence of oxygen and the surface Mo carbide will spontaneously oxidize, making it difficult to quantify the adsorbed H by H_2-O_2 titration.

Therefore, we chose to quantitatively analyze the Pt cluster size by CO chemical adsorption.

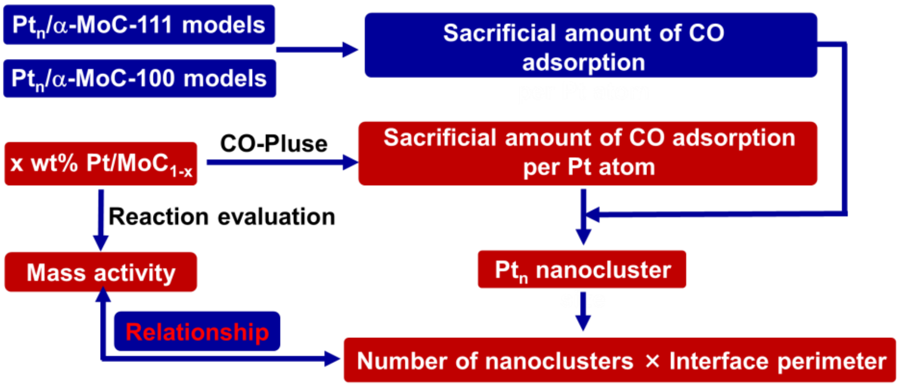

Fig. S1 The research concept diagram. The main implication is the method for determining Pt_n nanocluster size through CO adsorption, using a combined DFT (blue) and experimental (red) approach. The parameter 'sacrificial amount of CO adsorption per Pt atom' is defined as the decrease in the number of CO molecules adsorbed on the α-MoC_{1-x} surface following the deposition of a Pt atom on the α-MoC_{1-x} surface.

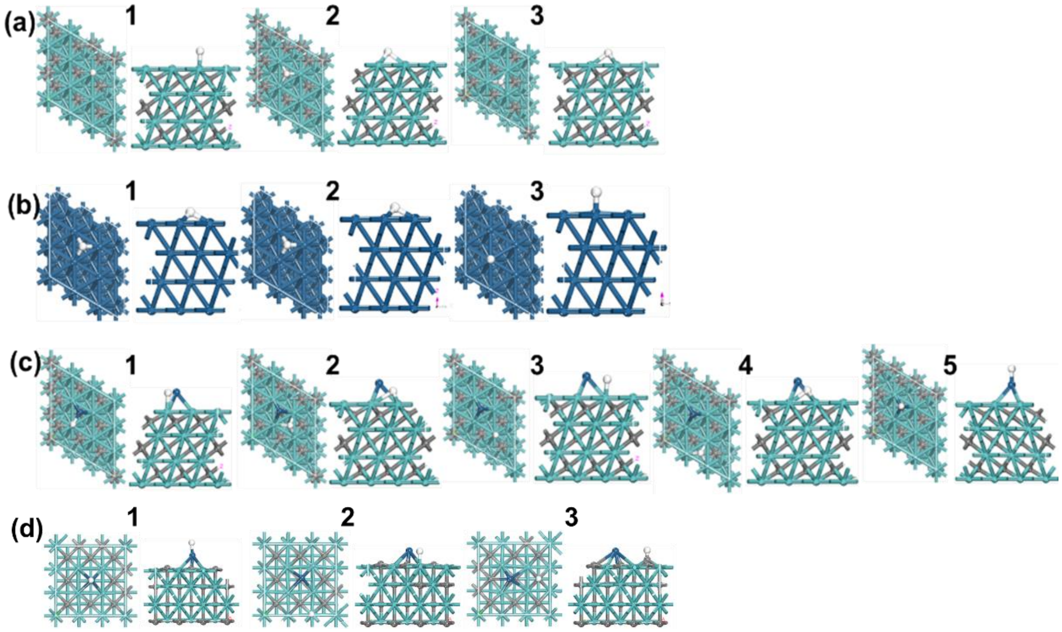

Fig. S2 Adsorption configurations of H atom on three models. (a) α-MoC-111, (b) Pt-111, (c) Pt_1/α-MoC-111, (d) Pt_1/α-MoC-100. Pt(blue color), Mo(green color), C(grey color), H(white color).

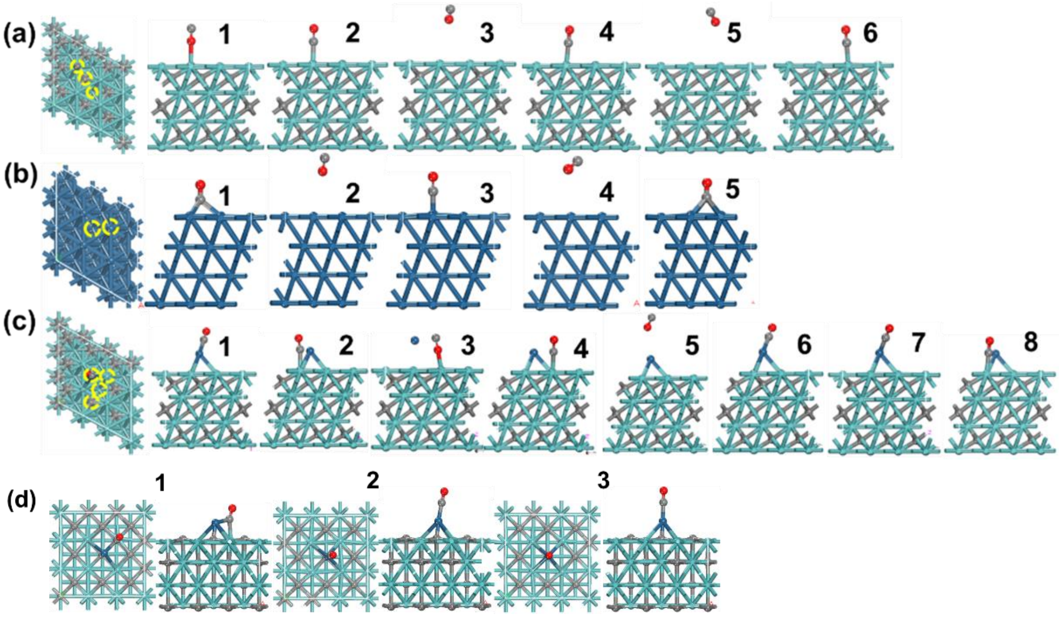

Fig. S3 Adsorption configurations of CO molecule on three models. (a) α-MoC-111, (b) Pt-111, (c) Pt_1/α-MoC-111, (d) Pt_1/α-MoC-100. Pt(blue color), Mo(green color), C(grey color), O(red color).

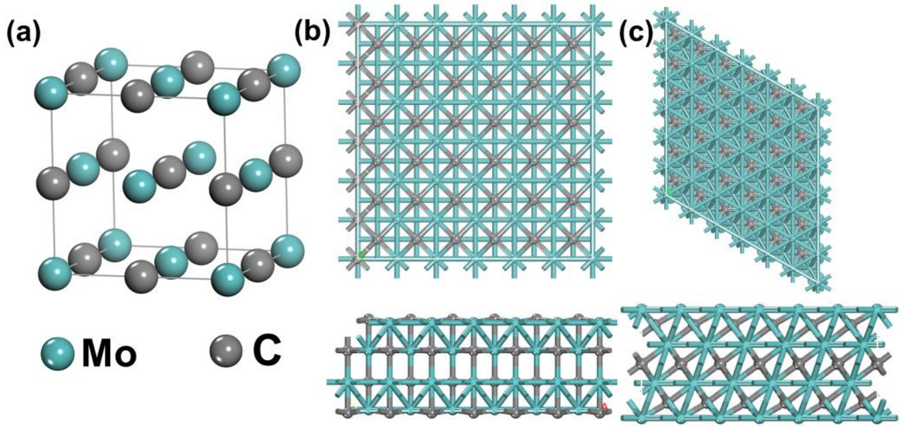

Fig. S4 Models of α-MoC. (a) α-MoC crystal cell; (b) α-MoC-100 model; (c) α-MoC-111 model. Mo(green color), C(grey color).

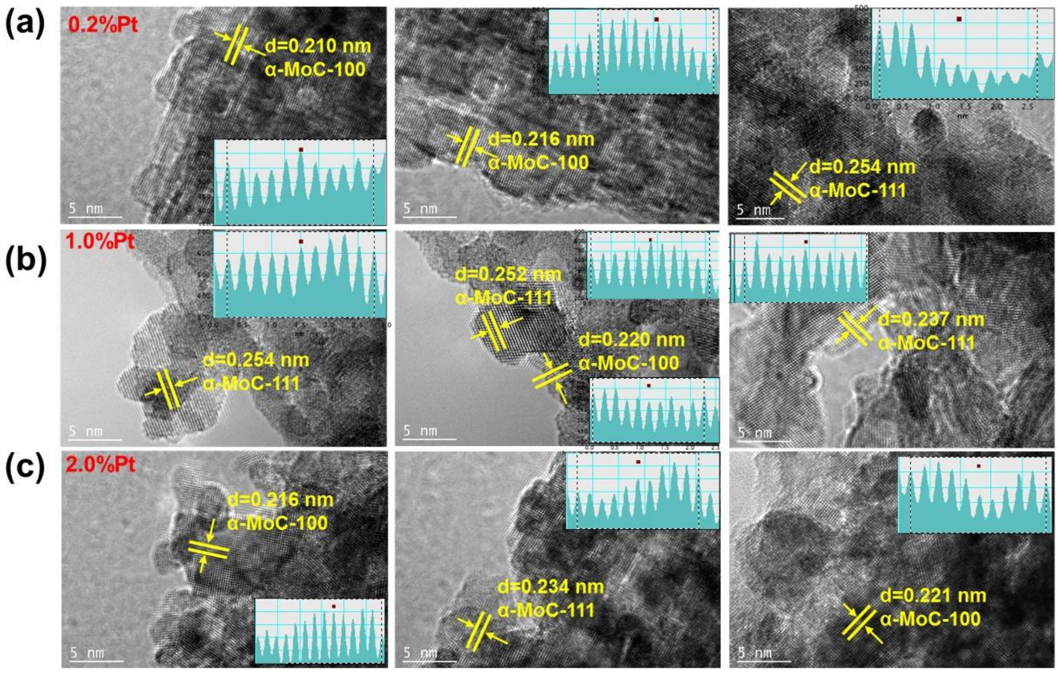

Fig. S5 HRTEM images of the Pt/α-MoC$_{1-x}$ catalysts. (a) 0.2% Pt/α-MoC$_{1-x}$; (b) 1.0% Pt/α-MoC$_{1-x}$; (c) 2.0% Pt/α-MoC$_{1-x}$.

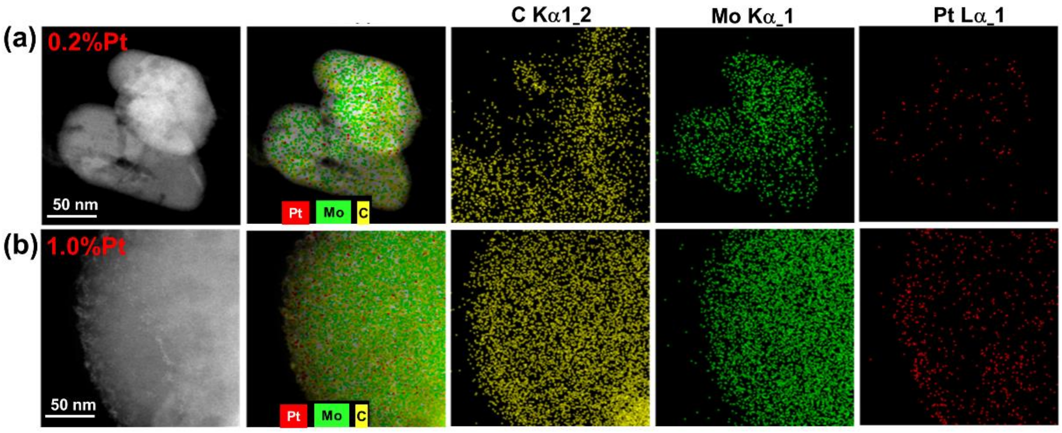

Fig. S6 EDS elemental mapping of the Pt/α-MoC$_{1-x}$ catalysts. (a) 0.2% Pt/α-MoC$_{1-x}$; (b) 1.0% Pt/α-MoC$_{1-x}$.

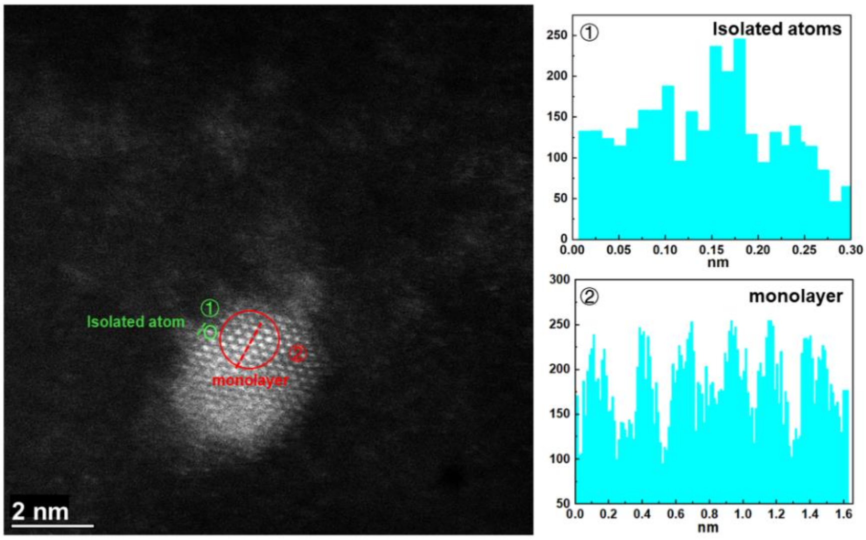

Fig. S7 The line-profile intensities of Pt atomic layers of 0.2% Pt/α-MoC_{1-x} sample.

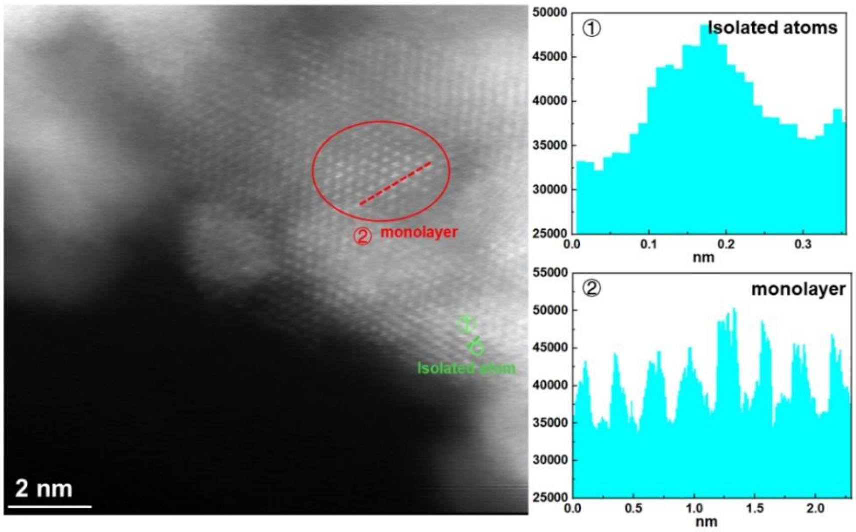

Fig. S8 The line-profile intensities of Pt atomic layers of 1.0% Pt/α-MoC$_{1-x}$ sample.

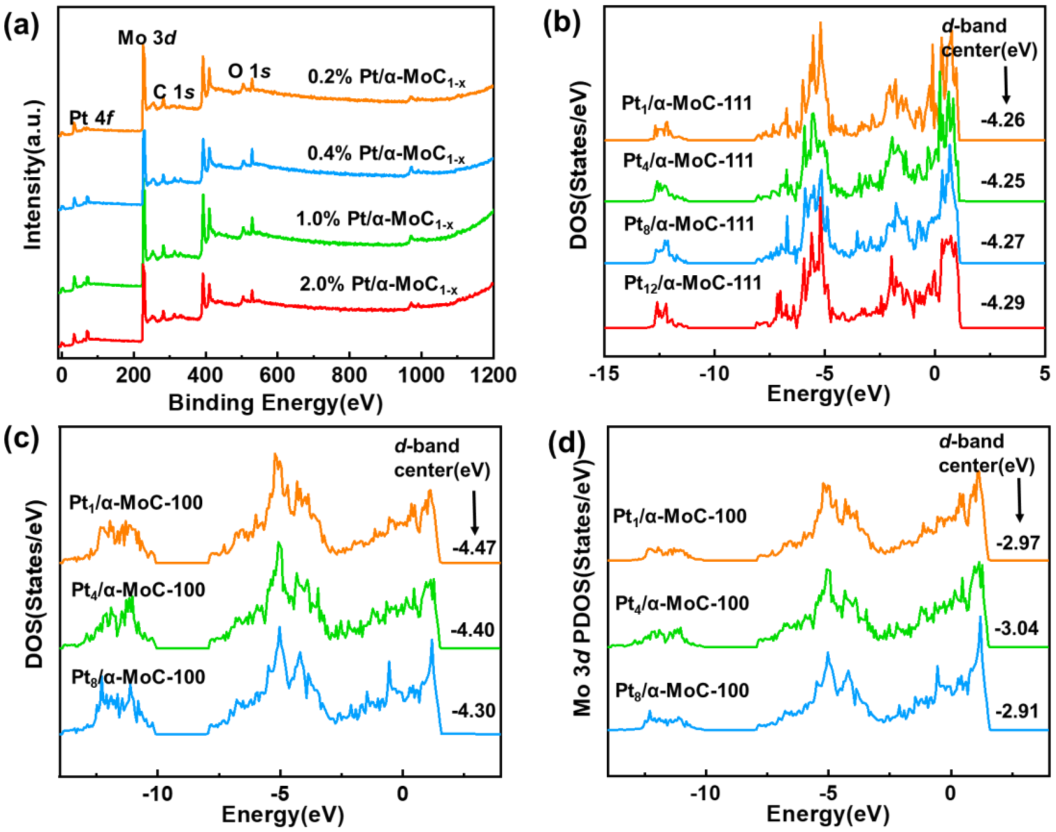

Fig. S9 Electronic properties of Pt/α-MoC catalysts and Pt/α-MoC models. (a) XPS survey spectra of Pt/α-MoC_{1-x} catalysts; (b) Density of states distribution of the Pt_1/α-MoC-111, Pt_4/α-MoC-111, Pt_8/α-MoC-111, Pt_{12}/α-MoC-111; (c) Density of states distribution of the Pt_1/α-MoC-100, Pt_4/α-MoC-100, Pt_8/α-MoC-100; (d) Partial densities of states (PDOSs) for the Mo atoms of Pt_1/α-MoC-100, Pt_4/α-MoC-100, Pt_8/α-MoC-100.

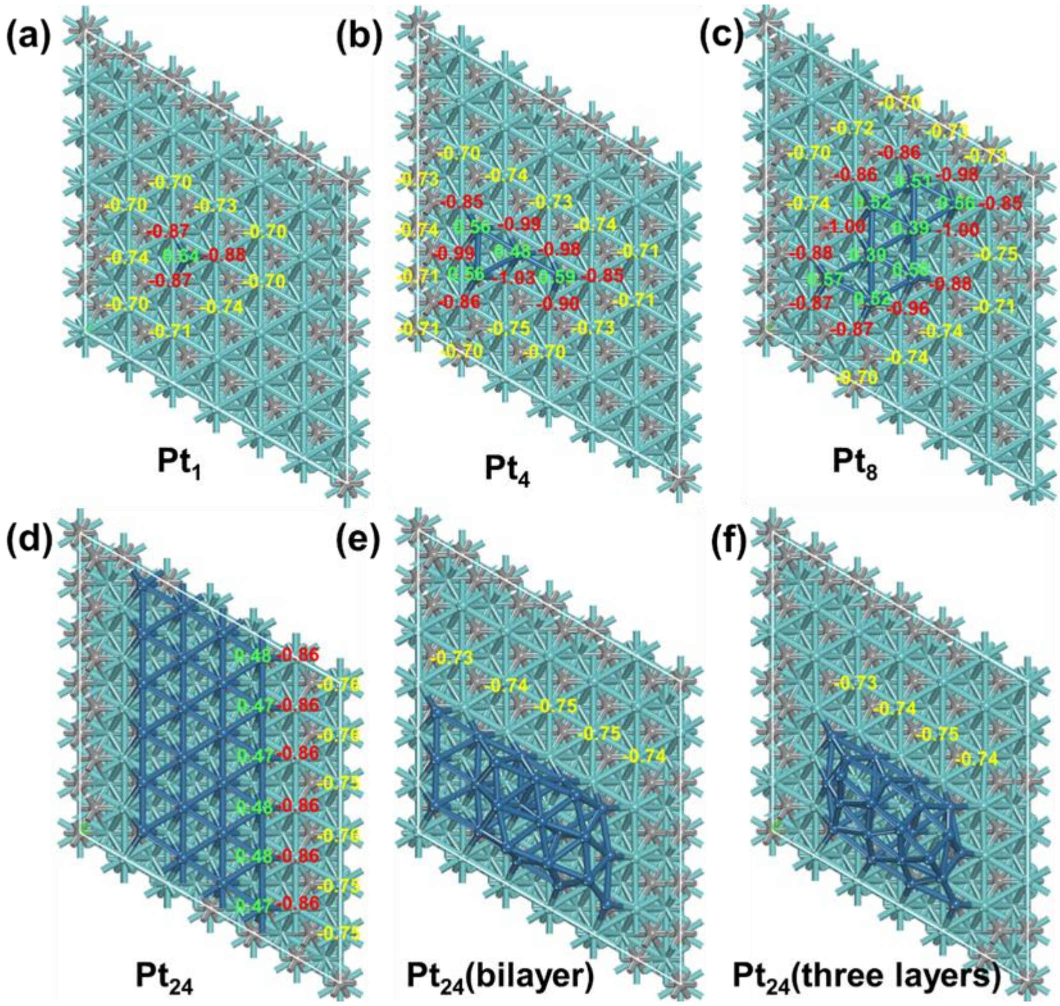

Fig. S10 Pt_n/α-MoC-111 model. (a) Pt_1, (b) Pt_4, (c) Pt_4, (d) Pt_{24} (monolayer), (e) Pt_{24} (bilayer), and (f) Pt_{24} (bilayer). The numerical value represents the charge of the corresponding atom. Pt(blue), Mo(green color), C(grey color)

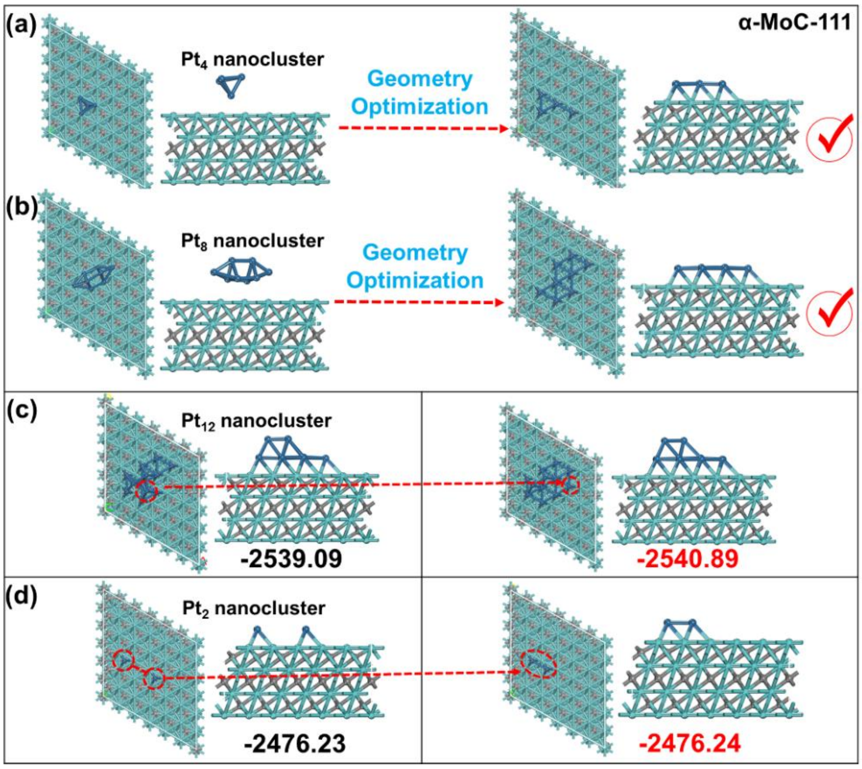

Fig. S11 The optimal configuration of Pt_n clusters on the α-MoC-111 surface. (a) Pt_4/α-MoC-111, (b) Pt_8/α-MoC-111, (c) Pt_{12}/α-MoC-111, (d) Pt_{12}/α-MoC-111. Pt(blue color), Mo(green color), C(grey color).

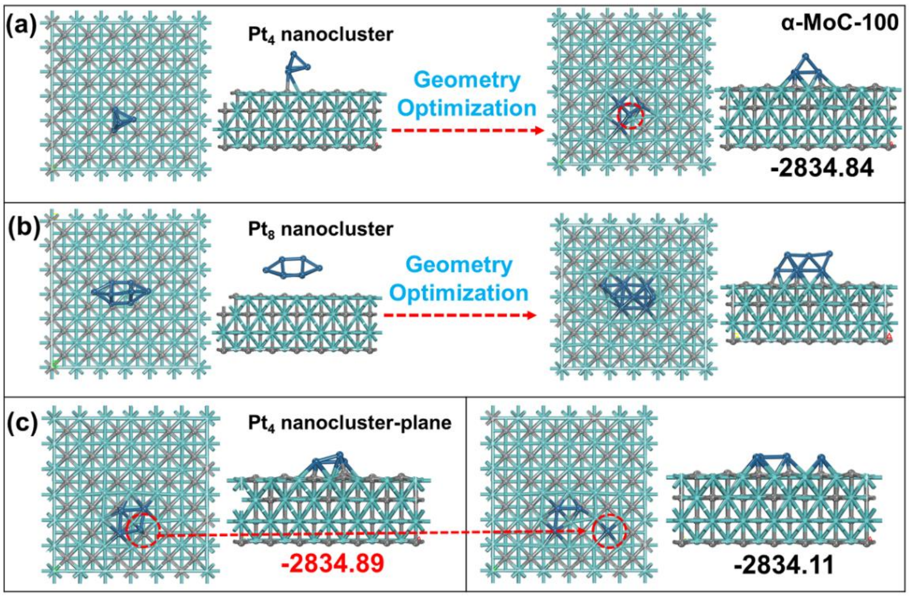

Fig. S12 The optimal configuration of Pt_n clusters on the α-MoC-100 surface. (a) Pt_4/α-MoC-100, (b) Pt_8/α-MoC-100, (c) Pt_4/α-MoC-111-plane. Pt(blue color), Mo(green color), C(grey color).

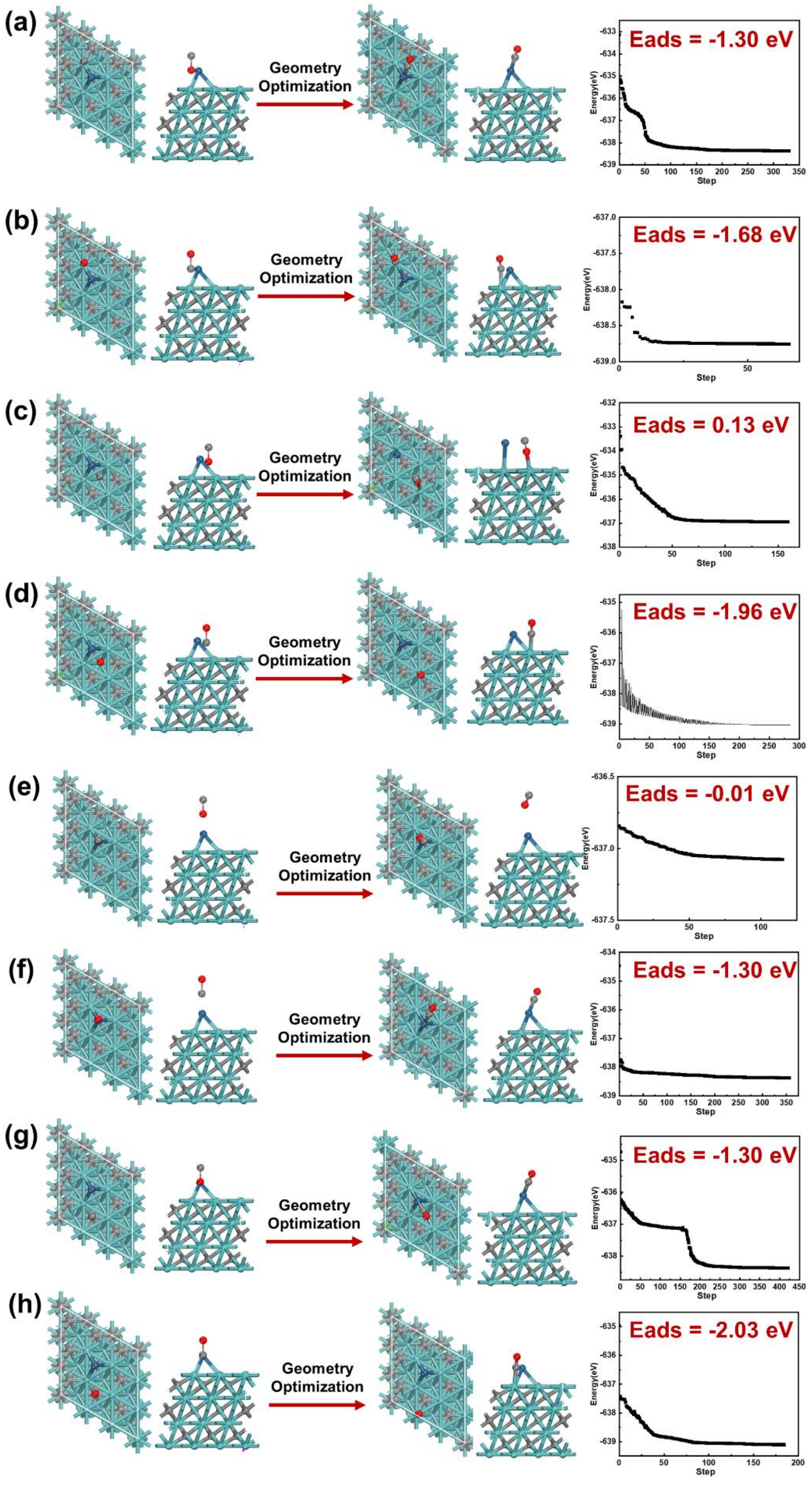

Fig. S13 Optimization process for different initial adsorption configurations of CO molecular on Pt$_1$/α-MoC-111 model. (a) C≡O···top-Mo, (b) O≡C···top-Mo, (c) C≡O···fcc-Mo(Pt), (d) O≡C···fcc-Mo(Pt), (e) C≡O···Pt, (f) O≡C···Pt, (g) C≡O···fcc-Mo, (h) O≡C···fcc-Mo. Pt(blue color), Mo(green color), C(grey color).

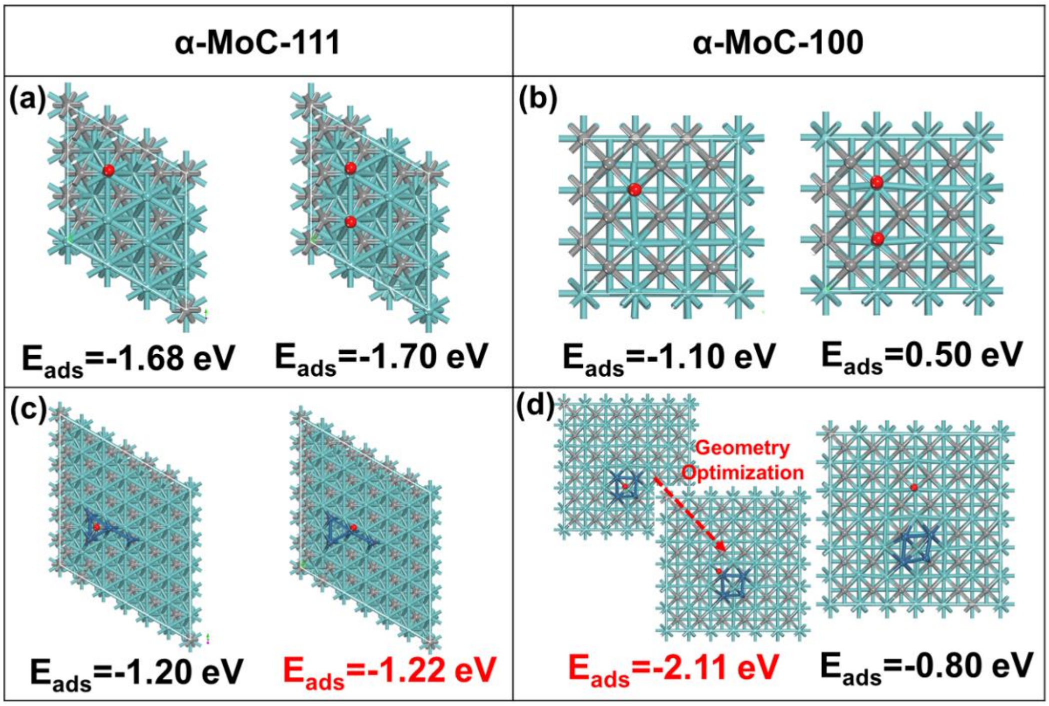

Fig. S14 Adsorption configurations of CO molecular on α-MoC models. (a) α-MoC-111, (b) α-MoC-100, (c) Pt_4/α-MoC-111, (d) Pt_4/α-MoC-100. Pt(blue color), Mo(green color), C(grey color).

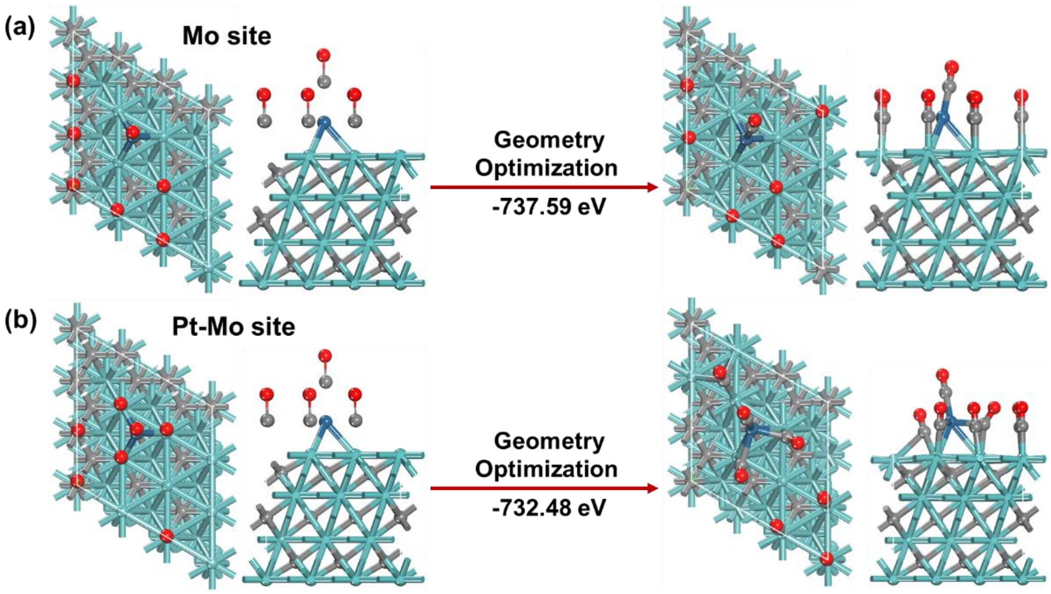

Fig. S15 The adsorption configuration of CO molecules. (a) Pt-MoC_{1-x} interface site, (b) non Pt-MoC_{1-x} interface site. Pt(blue color), Mo(green color), C(grey color). The CO adsorption on the Mo sites which bonded with Pt is not stable.

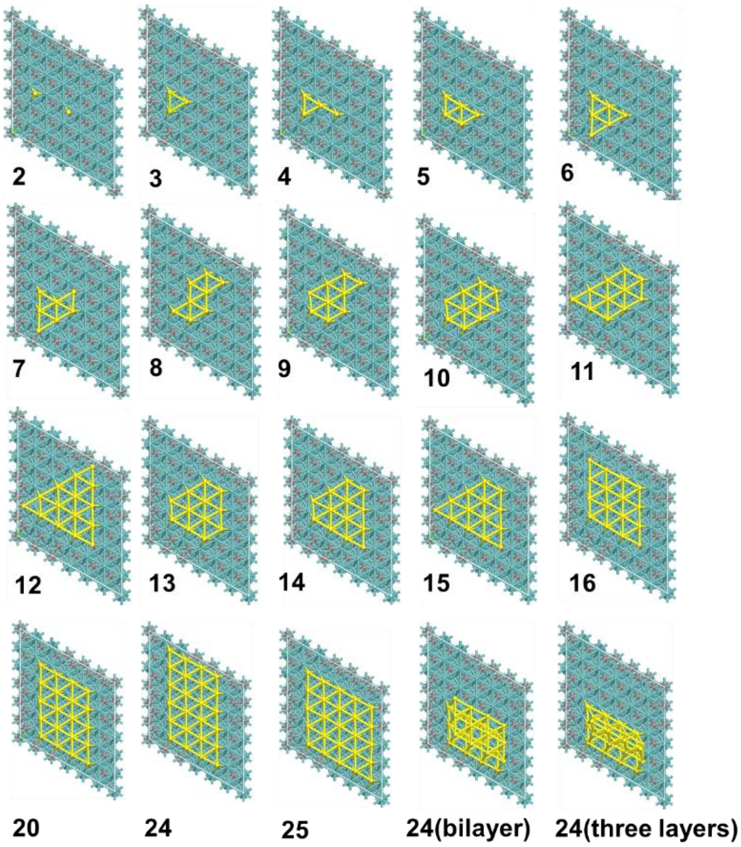

Fig. S16 The configurations of Pt_n/α-MoC-111 (n=2-25). Pt(yellow color), Mo(green color), C(grey color).

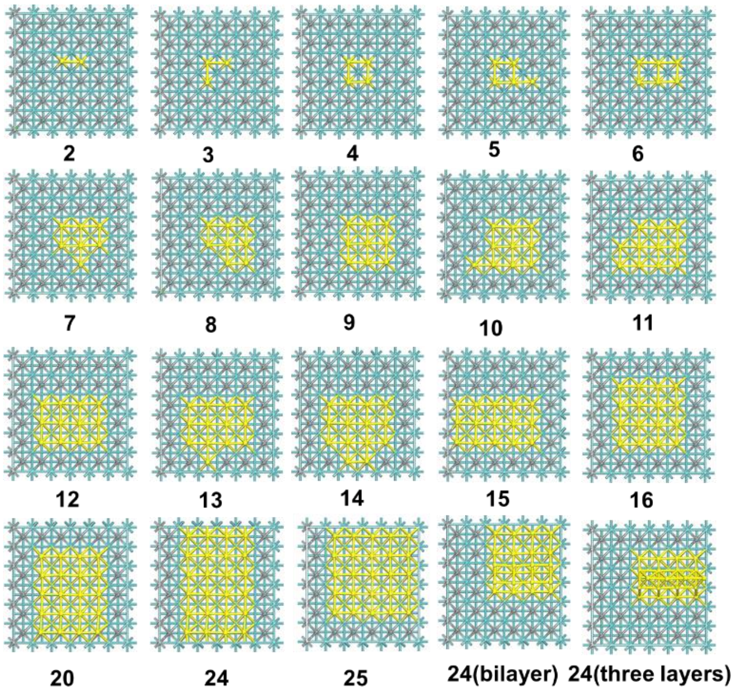

Fig. S17 The configurations of Pt_n/α-MoC-100 (n=2-25). Pt(yellow color), Mo(green color), C(grey color).

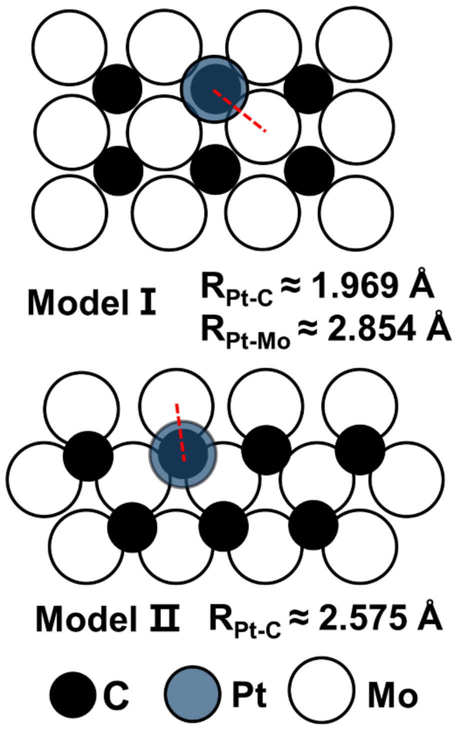

Fig. S18 The possible configurations of the isolated Pt anchored over the α-MoC surface with molybdenum or carbon as bridges. The feature of the structure when $R_{Pt-Mo} \approx 2.854$ Å and $R_{Pt-C} \approx 1.969$ Å, and the feature of the structure when $R_{Pt-Mo} \approx 2.576$ Å are included. In Model I, the Pt atom is located at the four-fold site of molybdenum and the top site of C atom on α-MoC-100 surface. In Model II, the Pt atom is also located at the three-fold site of surface molybdenum atom of α-MoC-111 surface.

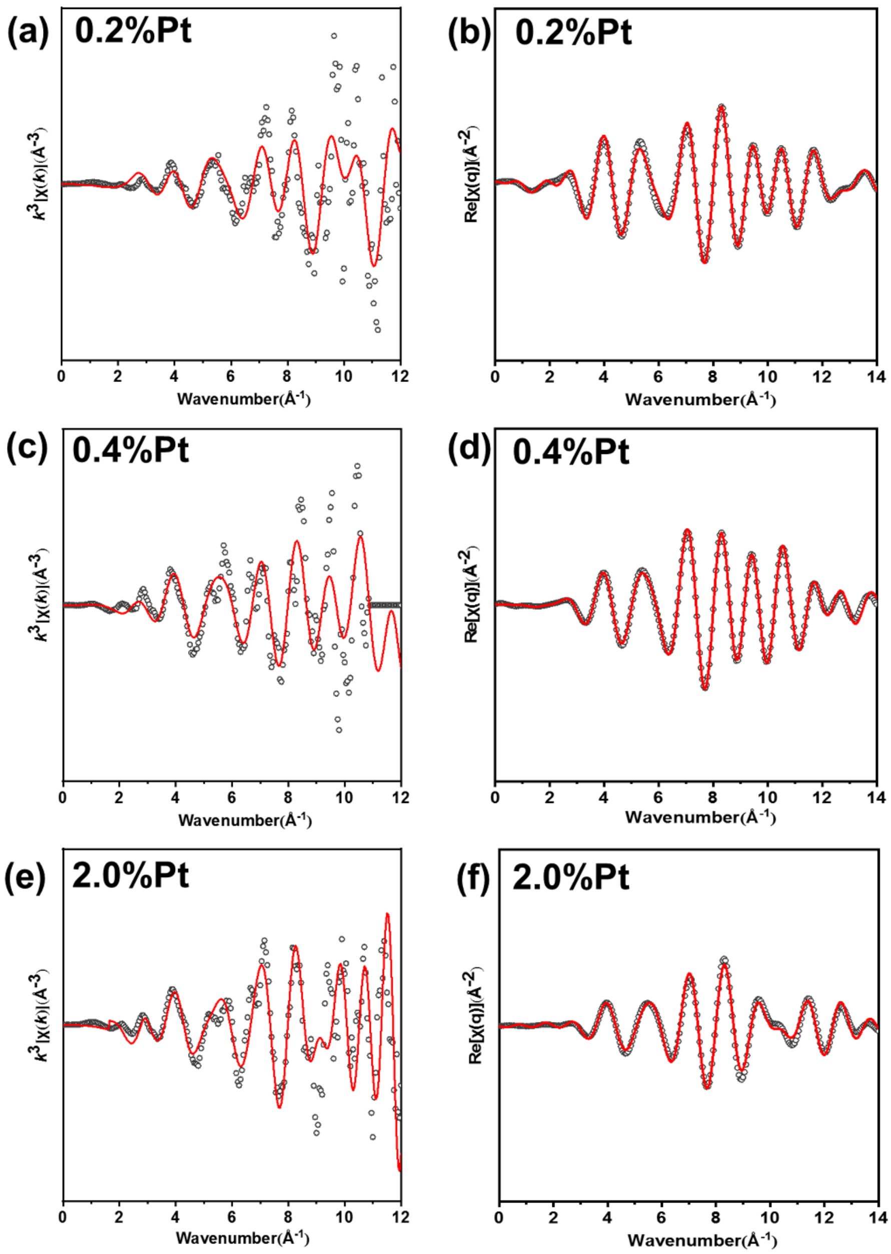

Fig. S19 EXAFS (points) and the curvefit (line) of Pt L_3-edge in k-space and q-space, respectively. (a-b) 0.2% Pt/α-MoC_{1-x}, (c-d) 0.4% Pt/α-MoC_{1-x}, (e-f) 2.0% Pt/α-MoC_{1-x}.

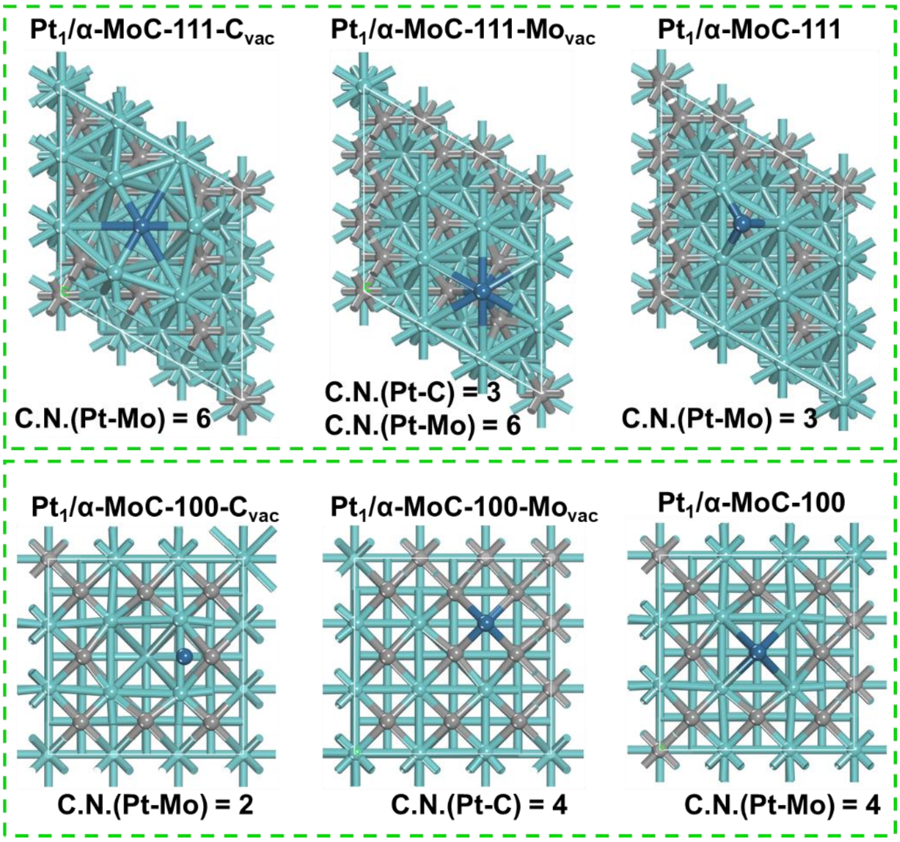

Fig. S20 The configurations of Pt on different surfaces. (Pt_1/α-MoC-111-C_{vac}, Pt_1/α-MoC-111-Mo_{vac}, Pt_1/α-MoC-111, Pt_1/α-MoC-100-C_{vac}, Pt_1/α-MoC-100-Mo_{vac}, Pt_1/α-MoC-100). C.N. represents the numbers of Pt-Mo bond or Pt-C bond. Pt(blue color), Mo(green color), C(grey color)

Table S1 Adsorption energies of H species on α-MoC-111, Pt -111, Pt_1/α-MoC-111, Pt_1/α-MoC-100 models

**Adsorbate**	**α-MoC-111**			**Pt-111**			**Pt_1/α-MoC-111**			**Pt_1/α-MoC-100**		
	**No.**	**Site**	**E_{ads} (eV)**	**No.**	**Site**	**E_{ads} (eV)**	**No.**	**Site**	**E_{ads} (eV)**	**No.**	**Site**	**E_{ads} (eV)**
**H**	1	top-Mo	-0.43	1	fcc-Pt	-0.44	1	top-Mo	-0.25	1	top-Pt	-0.35
	2	fcc-Mo	-0.50	2	fcc-Pt	-0.41	2	fcc-Mo	-0.26	2	top-Mo	0.22
	3	fcc-Mo	-0.74	3	top-Pt	-0.44	3	top-Mo	-0.37	3	top-C	-0.38
							4	fcc-Mo	-0.79			
							5	top-Pt	0.03			
**CO**	1	O-top-Mo	-0.06	1	fcc-Pt	-1.77	1	C-top-Pt	-1.30	1	C-top-Mo	-1.23
	2	C-top-Mo	-2.00	2	--	-0.004	2	C-top-Mo(Pt)	-1.68	2	C-top-Pt	-2.35
	3	--	3.82	3	top-Pt	-1.59	3	O-top-Mo	0.13	3	C-top-Pt	-2.31
	4	C-top-Mo	-1.98	4	--	-0.01	4	C-top-Mo	-1.96			
	5	--	-0.001	5	fcc-Pt	-1.71	5	--	-0.01			
	6	C-top-Mo	-1.98				6	C-top-Pt	-1.30			
							7	C-top-Pt	-1.30			
							8	C-top-Mo	-2.03			

Note: The data corresponds to Fig. S2 and Fig. S3, red markings indicate adsorption energy of the optimal configuration.

Table S2 Textural properties of the Pt/α-MoC_{1-x} catalysts with different loadings

Catalyst	Cell parameters (a, b, c, α, β, γ) [a]	BET surface area($cm^3 \cdot g^{-1}$) [b]	Single point Pore volume($cm^3 \cdot g^{-1}$) [b]	BJH Desorption Pore size(nm) [b]	Pt content (wt%) [c]
0.2% Pt/α-MoC_{1-x}	3.01,3.01,14.61 （90，90，120）	76.71	0.17	7.00	0.19
0.4% Pt/α-MoC_{1-x}	2.99，2.99，14.52 （90，90，120）	84.41	0.17	6.21	0.41
1.0% Pt/α-MoC_{1-x}	2.99，2.99，14.52 （90，90，120）	72.83	0.17	6.93	1.07
2.0% Pt/α-MoC_{1-x}	2.99, 2.99, 14.52 （90，90，120）	71.11	0.16	7.08	2.10

[a] Determined by XRD. [b] Determined by BET. [c] Determined by ICP-AES.

Table S3 The sacrificial CO adsorption per Pt atom of Pt/α-MoC_{1-x} catalysts with different loadings (Data from the CO-pulse experiment)

Catalyst	Number of Pt atoms(g^{-1})	Accumulated adsorption capacity of CO ($cm^3 \cdot g^{-1}$)	Accumulated adsorption number of CO (g^{-1})	Sacrificial CO adsorption per Pt atom
0.0 % Pt/α-MoC_{1-x}	0	0.78	2.10E+19	
0.2% Pt/α-MoC_{1-x}	5.86E+18	0.53	1.43E+19	-1.14
0.4% Pt/α-MoC_{1-x}	1.26E+19	0.41	1.11E+19	-0.78
0.7% Pt/α-MoC_{1-x}	2.16E+19	0.22	5.96E+18	-0.69
1.0% Pt/α-MoC_{1-x}	3.30E+19	0.12	3.27E+18	-0.54
2.0% Pt/α-MoC_{1-x}	6.48E+19	0.32	8.68E+18	-0.19
3.0% Pt/α-MoC_{1-x}	9.26E+19	0.41	1.11E+19	-0.11

Nuber of Pt atoms = Loading $\times N_A \div 195.08$

Accumulated adsorption number of CO = Accumulated adsorption capacity of CO $\times N_A \div (22.4 \times 10^3)$

Sacrificial CO adsorption per Pt atom = (Accumulated adsorption capacity of CO (x% Pt) - Accumulated adsorption capacity of CO (0.0% Pt)) $\div$ Number of Pt atoms

Table S4 The sacrificial CO adsorption per Pt atom of Pt_n/α-MoC-111 models (n=1-48)

Number of Pt atoms	Sacrificial CO	Increased amount	Changes in the	Sacrificial CO	Interface

	adsorption per Pt atom at Mo sites	of CO adsorption sites at Pt sites	number of CO adsorption sites	adsorption per Pt atom	perimeter per Pt_n nanocluster
1(monolayer)	3	1	-2	-2	9
2(monolayer)	5	2	-3	-1.5	11
3(monolayer)	6	3	-3	-1	12
4(monolayer)	8	4	-4	-1	12
5(monolayer)	9	5	-4	-0.8	14
6(monolayer)	10	6	-4	-0.67	15
7(monolayer)	12	7	-5	-0.71	16
8(monolayer)	14	8	-6	-0.75	17
9(monolayer)	15	9	-6	-0.67	17
10(monolayer)	16	10	-6	-0.6	17
11(monolayer)	18	11	-7	-0.64	18
12(monolayer)	19	12	-7	-0.58	18
13(monolayer)	21	13	-8	-0.62	19
14(monolayer)	23	14	-9	-0.64	20
15(monolayer)	25	15	-10	-0.67	21
16(monolayer)	24	16	-8	-0.5	22
19(monolayer)	29	19	-10	-0.53	22
19(bilayer)	19	15	-4	-0.16	18
20(monolayer)	29	20	-9	-0.45	23
24 (monolayer)	34	24	-10	-0.42	26
24(bilayers)	19	16	-3	-0.12	20
24(three layers)	19	20	-1	-0.04	19
25(monolayer)	35	25	-10	-0.4	25
48(monolayer)	62	48	14	-0.29	37
48(bilayers)	34	30	-4	-0.08	25

Changes in the number of CO adsorption sites = Increased amount of CO adsorption sites at Pt sites - Sacrificial CO adsorption per Pt atom at Mo sites

Sacrificial CO adsorption per Pt atom = Changes in the number of CO adsorption sites ÷ Number of Pt atoms

Note: A negative value for “Sacrificial CO adsorption per Pt atom” parameter indicates that the amount of CO adsorbed is decreasing, while a positive value means that the amount of CO adsorbed is increasing.

Table S5 The sacrificial CO adsorption per Pt atom of Pt_n/α-MoC-100 models (n=1-48)

Number of Pt atoms	Sacrificial CO adsorption per Pt atom at Mo sites	Increased amount of CO adsorption sites at Pt sites	Changes in the number of CO adsorption sites	Sacrificial CO adsorption per Pt atom	Interface perimeter per Pt_n nanocluster
1(monolayer)	4	1	-3	-3	12
2(monolayer)	6	2	-4	-2	14
3(monolayer)	8	3	-3	-1	15
4(monolayer)	9	4	-5	-1.25	16
5(monolayer)	11	5	-6	-1.2	16
6(monolayer)	12	6	-6	-1	18
7(monolayer)	14	7	-7	-1	19
8(monolayer)	15	8	-7	-0.87	20
9(monolayer)	16	9	-7	-0.78	20
10(monolayer)	18	10	-8	-0.8	22
11(monolayer)	19	11	-8	-0.73	22
12(monolayer)	20	12	-8	-0.67	22
13(monolayer)	22	13	-9	-0.69	24
14(monolayer)	23	14	-9	-0.64	24
15(monolayer)	24	15	-9	-0.6	24
16(monolayer)	25	16	-9	-0.56	24
20(monolayer)	30	20	-10	-0.5	26
25(monolayer)	36	25	-11	-0.44	28
24 (monolayer)	35	24	-11	-0.46	28
24(bilayers)	25	20	-5	-0.21	24
24(three layers)	20	20	0	0	22
48(monolayer)	63	48	-15	-0.31	40
48(bilayers)	35	28	-7	-0.15	24

Table S6 EXAFS fitting results of the Pt/α-MoC_{1-x} catalysts with different loadings

Catalysts	Shell(Path)	Bond length (Å)	ΔR(Å)	C.N.	E_0 shift (eV)	σ^2(Å^2)
0.2% Pt/α-MoC_{1-x}	Pt-C(Model I)	1.969	-0.16±0.06	2	4.2	0.011±0.008
	Pt-Mo(Model II)	2.576	-0.03±0.07	3		0.005±0.023
	Pt-Mo(Model I)	2.854	-0.10±0.09	2		0.004±0.011
0.4% Pt/α-MoC_{1-x}	Pt-C(Model I)	1.969	-0.02±0.03	2.9	1.2	0.006±0.003
	Pt-Mo(Model II)	2.576	+0.12±0.01	2		0.002±0.001
	Pt-Mo(Model I)	2.854	-0.08±0.03	3		0.013±0.005
2.0% Pt/α-MoC_{1-x}	Pt-C(Model I)	1.969	+0.09±0.06	2	10	0.003±0.002
	Pt-Mo(Model II)	2.576	-0.10±0.02	2		0.001±0.022
	Pt-Mo(Model I)	2.854	-0.02±0.02	3		0.008±0.002
	Pt-Pt(Pt)	2.788	+0.02±0.01	4.8		0.004±0.002

Table S7 Interface perimeter per gram of catalyst of the Pt/α-MoC_{1-x} catalysts with different loadings

Loading (%)	Sacrificial CO adsorption per Pt atom		Nanocluster size (Number of Pt atoms)	Number of Pt_n nanoclusters	Interface perimeter per Pt_n nanocluster	Interface perimeter per gram of catalyst (g_{cat}^{-1})	Mass activity ($mmol_{H2}\ g_{cat}^{-1}\ h^{-1}$)
	CO-pulse experiment	Model					
			**α-MoC-111**				
0.2%	-0.99	-1	4(monolayer)	1.54E+18	12	2.04E+19	13.33
0.4%	-0.78	-0.80	5(monolayer)	2.47E+18	14	3.54E+19	31.84
0.7%	-0.70	-0.71	7(monolayer)	2.70E+18	16	4.32E+19	44.33
1.0%	-0.56	-0.58	12(monolayer)	2.57E+18	18	4.73E+19	56.63
2.0%	-0.16	-0.16	19(bilayers)	3.25E+18	18	5.94E+19	103.04
			**α-MoC-100**				
0.2%	-0.99	-1	6(monolayer)	1.03E+18	15	2.04E+19	13.33
0.4%	-0.78	-0.78	9(monolayer)	1.23E+18	20	2.84E+19	31.84
0.7%	-0.70	-0.69	13(monolayer)	1.66E+18	24	3.99E+19	44.33
1.0%	-0.56	-0.56	16(monolayer)	1.93E+18	24	4.72E+19	56.63
2.0%	-0.16	-0.21	24(bilayers)	2.57E+18	24	6.27E+19	103.04

Number of Pt_n nanoclusters = Total number of Pt atoms ÷ Nanocluster size (Number of Pt atoms)

Interface perimeter per gram of catalyst = Interface perimeter per Pt_n nanocluster × Number of Pt_n nanoclusters

Note: Nanocluster size and Interface perimeter per Pt_n nanocluster are obtained from Table S4 and Table S5

Table S8 Comparison of activation energy

Catalysis	Ea (kJ·mol^{-1})	Ref.
Rh_1/α-MoC_{1-x}	46.4±4.0	1
Pd_1/α-MoC_{1-x}	46.3±1.3	
Ir_1/α-MoC_{1-x}	43.0±2.3	
Pt_1/α-MoC_{1-x}	47.6±1.9	
Au_1/α-MoC_{1-x}	43.9±4.7	
α-MoC_{1-x}	60.0±5.3	
Pt/Al_2O_3	83	2
Mo_2C/Al_2O_3	68	
Mo_2C	63	
0.2 wt%Pt_1/α-MoC	38	3
2 wt%(Pt_1-Pt_n)/α-MoC	31	
Ir_1/α-MoC	43.0±2.3	4
α-MoC	60.0±5.3	
α-MoC_{1-x}	64	5
2 wt%Au/α-MoC_{1-x}	22	
(0.9%)Au/a-MoC(leached by NaCN)	41	
Pt/Mo_2C	53	6
0.2 wt%Pt/α-MoC_{1-x}	62.2	Our results
0.4 wt%Pt/α-MoC_{1-x}	58.5	
1.0 wt%Pt/α-MoC_{1-x}	51.0	
2.0 wt%Pt/α-MoC_{1-x}	55.9	
α-MoC_{1-x}	75.2	

Supplementary References

1. Li J, Sun L, Wan Q, Lin J, Lin S, Wang X. Alpha-MoC supported noble metal catalysts

for water-gas shift reaction: single-atom promoter or single-atom player. *J. Phys. Chem. Lett.* **12**, 11415-11421 (2021).

2. Wang G, Schaidle JA, Katz MB, Li Y, Thompson LT. Alumina supported Pt–Mo_2C catalysts for the water–gas shift reaction. *J. Catal.* **304**, 92-99 (2013).
3. Zhang X, et al. A stable low-temperature H2-production catalyst by crowding Pt on α-MoC. *Nature* **589**, 396-401 (2021).
4. Sun L, et al. High-efficiency water gas shift reaction catalysis on α-MoC promoted by single-atom Ir species. *ACS Catal.* **11**, 5942-5950 (2021).
5. Yao S, et al. Atomic-layered Au clusters on α-MoC as catalysts for the low-temperature water-gas shift reaction. *Sci. Found. China* **357**, 389 (2017).
6. Schweitzer NM, Schaidle JA, Ezekoye OK, Pan X, Linic S, Thompson LT. High activity carbide supported catalysts for water gas shift. *J. Am. Chem. Soc.* **133**, 2378-2381 (2011).
