# Supplementary material for: Quantification and optimization of platinum–molybdenum carbide interfacial sites to enhance low-temperature water-gas shift reaction
Source: Nat Commun. 2025 Jan 28;16:1098. doi: 10.1038/s41467-025-55886-y (PMC11775272; doi:10.1038/s41467-025-55886-y)
Supplement: Supplementary file 2 — Description of Additional Supplementary Files [file 41467_2025_55886_MOESM2_ESM.pdf]

Description of Additional Supplementary Files

File Name: Supplementary Data 1
Description: The atomic coordinates of the optimized computational models were provided in this file.
